# Infarct core growth rate and 90-day outcomes in ischemic stroke: subgroup analysis based on onset-to-recanalization time

**DOI:** 10.3389/fneur.2025.1553357

**Published:** 2025-04-30

**Authors:** Sha Chen, Guofang Chen, Changzhi Zhao, Enle Wang, Yewen Zhou, Manhua Ding, Yang Zhang

**Affiliations:** ^1^Jiangsu Key Laboratory of New Drug Research and Clinical Pharmacy, Xuzhou Medical University, Xuzhou, China; ^2^Department of Neurosurgery, Xuzhou Center Hospital, Xuzhou Clinical School of Xuzhou Medical University, Southeast University Affiliated Xuzhou Central Hospital, Xuzhou, China; ^3^Department of Neurology, Xuzhou Center Hospital, Xuzhou Clinical School of Xuzhou Medical University, Southeast University Affiliated Xuzhou Central Hospital, Xuzhou, China; ^4^Southeast University School of Medicine, Southeast University School, Nanjing, China; ^5^Department of Radiotherapy, Xuzhou Cancer Hospital, Xuzhou, China; ^6^Department of Neurology and Clinical Research Center of Neurological Disease, The Second Affiliated Hospital of Soochow University, Suzhou, China

**Keywords:** infarct core growth rate, infarct core volume, onset-to-recanalization time, endovascular treatment, outcome

## Abstract

**Background:**

It is essential to understand the factors that influence patient outcomes in stroke research. The infarct core growth rate (ICGR) is emerging as a potentially valuable marker, but its relationship with patient outcomes, especially concerning the onset-to-recanalization time (ORT), requires further clarification. This study investigates the impact of ICGR on 90-day (90d) outcomes in acute ischemic stroke patients and explores whether stratifying ICGR analysis based on ORT provides more detailed prognostic insights.

**Methods:**

This study retrospectively analyzed patients with acute ischemic stroke with anterior circulation large vessel occlusion (AIS-ACLVO) who underwent endovascular treatment (EVT) between January 2021 and December 2023. Their clinical characteristics, baseline and imaging data were recorded upon admission. Clinical outcomes were evaluated using the modified Rankin Scale (mRS) at 90 days post-procedure. The least absolute shrinkage and selection operator (LASSO) regression was employed for data screening. Multivariable logistic regression analysis was performed to explore the relationship between ICGR and 90-day (90d) clinical outcome. Additionally, a stratified analysis based on ORT was conducted to compare the diagnostic performance of ICGR and infarct core volume (ICV) at different time points.

**Results:**

A total of 153 patients were included in the analysis. Univariate and Lasso regression analyses showed that the group with unfavorable outcomes had statistically significant differences in ICGR, age, history of atrial fibrillation, history of drinking, admission blood glucose level, Alberta Stroke Program Early CT Score (ASPECTS), and National Institutes of Health Stroke Scale (NIHSS) score compared to the favorable outcome group (all *p* < 0.05). Furthermore, multivariate logistic regression analysis indicated that ICGR was independently associated with clinical outcome in AIS-ACLVO patients [Odds Ratio (OR) 1.101, 95% confidence interval (CI) 1.029–1.178; *p* = 0.005]. When stratified by median ORT, the ICGR remained a strong predictor of outcome within 8 h (OR 1.188, 95% CI 1.048–1.347; *p* = 0.007), and proved to be a better predictor than ICV [area under the Receiver Operating Characteristic (AUROC) curve, 0.816 vs. 0.750, *p* = 0.024].

**Conclusion:**

Our research indicates that the ICGR correlates with 90d clinical outcomes in AIS-ACLVO patients: a faster rate is associated with poorer outcomes. Within 8 h of ORT, the ICGR serves as a better predictor of 90d outcome than ICV.

## Introduction

The global incidence of stroke has been increasing in recent years (1). Acute ischemic stroke with anterior circulation large vessel occlusion (AIS-ACLVO) is one subtype of acute ischemic stroke that generally has a poorer prognosis (2). Research has shown that administering endovascular thrombectomy (EVT) within 24 h after the onset of symptoms, as opposed to relying on medical treatment alone, can significantly improve both short-term and long-term outcomes for most patients, while also reducing mortality rates (3–6). Nonetheless, a considerable percentage of patients—between 40% and 60%—still face poor outcomes (7, 8).

For patients with AIS-ACLVO, computed tomography perfusion (CTP) imaging has been widely utilized to identify reversible ischemic regions (ischemic penumbra) and irreversible ischemic regions (infarct core) (9). Yu et al. (10) used whole-brain CTP to calculate the optimal threshold for identifying the ischemic core in Chinese individuals, establishing that a relative cerebral blood flow (rCBF) of 30% or less, with a delay time of 3 s or more, is indicative of this condition. However, despite using the infarct core volume (ICV) and clinical characteristics as guidelines for surgical selection and individualized treatment for patients experiencing stroke due to large vessel occlusion, there was still an inaccurate prediction of prognosis for some patients.

Patients exhibiting identical ICV may experience disparate outcomes, depending upon the temporal discrepancy between the onset of symptoms and the restoration of blood flow in the impacted vessel (11). The rate of infarction, in synergy with the magnitude of the ICV and the interval from the onset of stroke to the acquisition of imaging, provides a holistic representation of the evolution of cerebral infarction. Infarct core growth rate (ICGR) = ICV (rCBF ≤ 30%)/time from symptom onset to imaging. ICGR has emerged as a critical metric for evaluating EVT efficacy. Alebers et al. (12) hypothesized that slower core growth dynamics might explain the superior outcomes of EVT over conservative management in late time-window clinical trials. This mechanistic insight has led to the clinical consensus that patients with rapid ICGR derive limited benefit from EVT.

The rapid ICGR defined in previous studies typically ranges from > 5–15 mL/h (12–14). Using a linear infarct core expansion model, one study demonstrated that patients with target mismatch and baseline ICV < 70 mL within early time windows exhibited significantly faster ICGR (>25 mL/h) compared to previously reported values (15). Intriguingly, these individuals with accelerated ICGR derived greater absolute benefit from EVT than thrombolysis-alone groups (9). However, consistent with prior investigations, this therapeutic advantage was restricted to specific populations characterized by relatively small baseline ICV and favorable imaging profiles.

To date, no consensus exists on the definition of rapid ICGR, and its impact on EVT outcomes remains controversial. This study aims to investigate the relationship between ICGR and the outcomes of patients at a 90-day follow-up after EVT.

## Methods

### Study design

This research was a single-center retrospective analysis, and consecutively included patients diagnosed with AIS-ACLVO who were seen in the outpatient or emergency green channel of our hospital from January 2021 to December 2023. All patients undergoing intravenous thrombolysis or EVT received written informed consent from the patient or their authorized representative prior to treatment. The study was approved by the Ethics Committee of Xuzhou Central Hospital. Demographic, clinical, and imaging data were prospectively collected and analyzed to evaluate treatment outcomes. Original datasets supporting this study’s conclusions are accessible from the corresponding author upon reasonable request.

### Patients population

The inclusion criteria for our study were as follows: (1) patients had acute stroke symptoms; (2) all patients underwent baseline imaging assessment, including noncontrast computed tomography (NCCT), CT angiography (CTA), and CT perfusion (CTP) scans; (3) CTA confirmed occlusion of the cervical internal carotid artery, as well as the M1 segment or proximal portion of the M2 segment of the middle cerebral artery; (4) age ≥ 18 years; (5) preoperative NIHSS score ≥ 6 upon admission; (6) onset-to-door time ≤ 24 h; (7) ICV ≤ 100 mL. The exclusion criteria were as follows: (1) CT scan of the brain showed hemorrhage; (2) occlusion of the posterior circulation or involvement of both circulations; (3) mRS score ≥ 2 before the onset of the disease; (4) severe dysfunction of important organs such as the heart, lungs, liver, and kidneys, with an expected life span of less than 6 months; (5) the time from symptom onset to imaging examination is unclear and imaging data is incomplete or unreadable; (6) expanded Thrombolysis in Cerebral Infarction (eTICI) grade < 2b50 after EVT (16); (7) patients who have not completed follow-up. Collected baseline data on the patients, including age, gender, vascular risk factors, blood pressure at admission, laboratory tests, stroke etiological classification, imaging studies, and time intervals.

### Imaging protocol and analysis

Imaging was conducted utilizing a Toshiba Aquilion One Vision 64-slice spiral CT scanner. CT parameters were 80 kV, 300 mA, 0.5 mm slice thickness, 0.5 mm slice interval, 220 × 220 mm field of view, 160 mm detector collimation, and 0.75 s/r gantry rotation speed. 50 mL of iopromide contrast agent was injected via the cubital vein at 4.0 mL/s, followed by a 30 mL saline flush at 4.0 mL/s. Dynamic volume scanning was performed with a 7 s delay. NCCT and CTA raw data were uploaded to a Shu-kun workstation, and CTP data were uploaded to iStroke 3.15 software for automated processing using a convolution algorithm. The software automatically calculated the ICV (rCBF < 30%), Ischemic volume (time to maximum, a key CTP parameter measuring when blood contrast reaches a specific brain region and peaks > 6 s), and mismatch volume (ischemic volume—ICV). Two experienced neurologists with over 5 years of experience jointly interpreted the images and calculated the Alberta Stroke Program Early CT Score (ASPECTS) (17), and disagreements were resolved through negotiation.

### EVT procedure

Clinical symptoms and imaging assessments were performed on AIS-ACLVO patients. For patients with an onset-to-door time of ≤4.5 h who were free of contraindications and had obtained informed consent, 0.9 mg/kg alteplase was administered intravenously for thrombolysis (18). The standard approach is to select the right femoral artery as the puncture site. After the puncture is successful, a cerebral angiography is conducted to identify the precise location of vascular occlusion and evaluate the status of collateral circulation. The American Society of Interventional and Therapeutic Neuroradiology/Society of Interventional Radiology (ASITN/SIR) grading system is employed for assessment (19). Then, a thrombus aspiration system was introduced and placed near the proximal occluded vessel. Under the guidance of the roadmap, a microcatheter and microguidewire were used to enter the occluded area. The microguidewire was then withdrawn, and a suitable-sized stent was placed to perform stent thrombectomy.

### Outcome evaluation

Patients were followed up via phone or outpatient clinic, after 90-day (90d) following EVT, by the same experienced neurologist using mRS for outcome assessment. A score of 0–2 was defined as a favorable outcome, while a score of 3–6 was defined as an unfavorable outcome, with a score of 6 signifying death (3).

### Statistical analysis

Statistical analysis was carried out with the help of IBM SPSS Statistics 27.0 and R 4.4.2 software. Continuous quantitative data adhering to a normal distribution were presented as $\bar{x}$ ± s, and group comparisons were conducted using an independent samples t-test. Data with non-normal distributions were reported as median and interquartile range (IQR), and the Mann–Whitney U test was used for comparison between the two groups. Count data was expressed as frequency and percentage, and the chi-square (χ^2^) test or Fisher’s exact test was used for comparison between the two groups. The “glmnet” package in R software was used to build the least absolute shrinkage and selection operator (LASSO) regression model and conduct data feature selection to reduce model collinearity and overfitting. Multivariate logistic regression analysis was used to identify the independent risk factors for unfavorable outcomes, and the odds ratios (OR) along with 95% confidence intervals (CI) were computed. The receiver operating characteristic (ROC) curve was plotted, and the area under the ROC curve (AUROC) was calculated. The DeLong test was used to evaluate the discriminatory power of different groups. It was considered statistically significant when the two-sided value of P was less than 0.05.

## Results

### Baseline characteristic of patients

**Figure 1:**
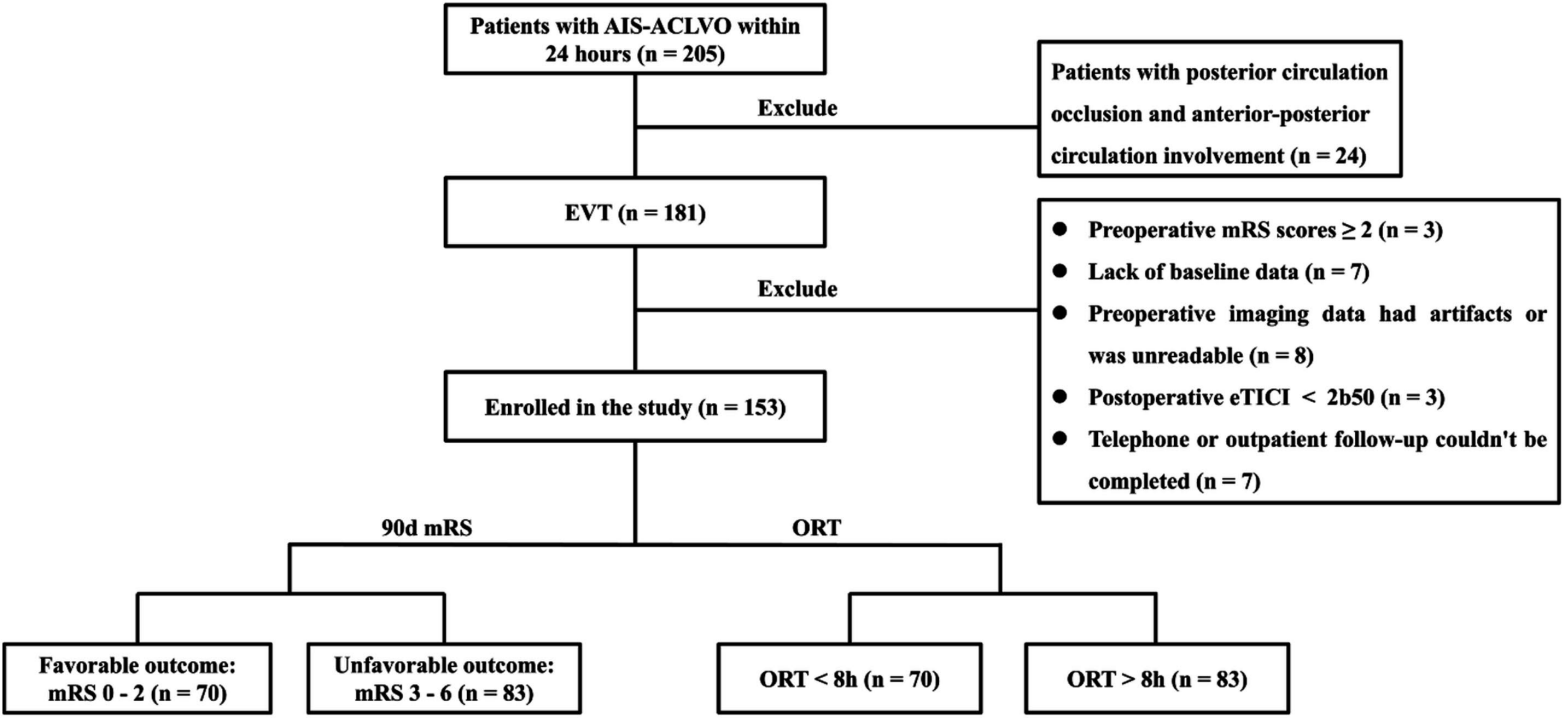
Patient selection flow-chart. AIS-ACLVO, Acute Ischemic Stroke with Anterior Circulation Large Vessel Occlusion; EVT, Endovascular Treatment; mRS, modified Rankin Scale; eTICI, expanded Thrombolysis in Cerebral Infarction; 90d, 90-day; ORT, Onset-to-recanalization time.

The research flowchart is shown in Figure 1. During the study, a total of 205 patients with AIS-LVO undergoing EVT were enrolled. After excluding 24 patients with involvement of both posterior and anterior circulations, 7 patients with incomplete baseline data, 3 patients with preoperative mRS score of ≥2, 8 patients with missing or degraded imaging data affecting interpretation, and 3 patients with postoperative eTICI grade <2b50, 153 patients were included in the subsequent analysis. There were 86 males (56.2%) with a median age of 70 (IQR 60, 77) years. 70 (45.8%) had a favorable prognosis, while 83 (54.2%) had an unfavorable outcome, including 36 deaths (23.5%).

### Univariate analysis and clinical feature selection

**Figure 2:**
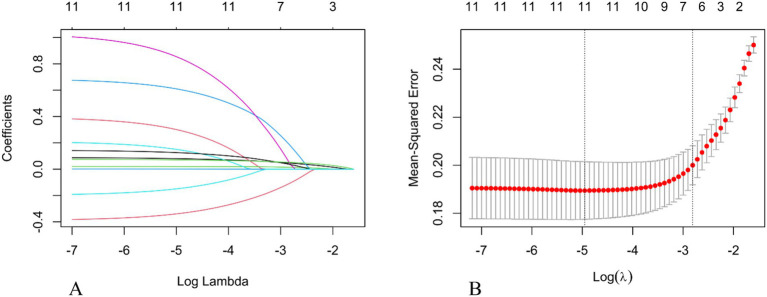
Selection of the optimal prognostic factors by LASSO regression analysis. **(A)** LASSO coefficient profiles of potential predictors. **(B)** Screening of the optimal penalization coefficient in the LASSO regression. LASSO, The Least Absolute Shrinkage and Selection Operator.

Univariate analysis revealed statistically significant differences between the unfavorable prognosis group and the favorable prognosis group in terms of age, gender, history of atrial fibrillation, coronary heart disease, history of alcohol consumption, admission blood glucose, ASPECTS score, NIHSS score, ASITN/SIR score, ICV, ischemic volume, and ICGR (all *p* < 0.05; see Table 1). Incorporating the aforementioned metrics into the Lasso regression model. Owing to the pronounced collinearity between the ICV and the ICGR, the ICV was omitted from the Lasso regression analysis. The findings indicated that gender, a history of coronary heart disease, ischemic volume, and the ASITN/SIR score were not retained in the model (Figure 2).

**Table 1:** Factors affecting outcomes in patients with AIS-ACLVO.

Indicator	All (*n* = 153)	Favorable (*n* = 70)	Unfavorable (*n* = 83)	Statistic	*p*-value
Demography					
Age (y; *M*, *IQR*)	70.00 (60.00,77.00)	64.00 (56.00,74.00)	73.00 (67.00,78.00)	−3.990	<0.001
Male [*n* (%)]	86 (56.2)	46 (65.7)	40 (48.2)	4.736	0.030
Vascular risk factors [*n* (%)]					
Atrial fibrillation	57 (37.3)	16 (22.9)	41 (49.4)	11.443	<0.001
Coronary heart disease	49 (32.0)	16 (22.9)	33 (39.8)	4.983	0.026
Smoking	31 (20.3)	19 (27.1)	12 (14.5)	3.782	0.052
Drinking	26 (17.0)	7 (10.0)	19 (22.9)	4.474	0.034
Baseline data					
SBP (mmHg^a^; *M*, *IQR*)	145.00 (131.50,165.00)	145.00 (130.00,165.00)	146.00 (134.00,168.00)	−0.704	0.482
DBP (mmHg^a^; *M*, *IQR*)	80.00 (75.00,90.00)	80.50 (75.00,87.75)	80.00 (75.00,90.00)	0.526	0.599
Admitted blood glucose (mmol/L; *M*, *IQR*)	7.27 (5.85,9.10)	6.92 (5.35,8.26)	7.66 (6.52,9.83)	−2.478	0.013
Preoperative NIHSS (*M*, *IQR*)	18.00 (12.00,24.00)	14.00 (8.00,19.00)	21.00 (17.00,26.00)	−5.209	<0.001
Radiologic characteristic					
ASPECTS (*M*, *IQR*)	8.00 (7.00,9.00)	9.00 (8.00,10.00)	8.00 (7.00,9.00)	2.976	0.003
ASITN/SIR (*M*, *IQR*)	1.75 (1.00,2.00)	2.00 (1.00,2.64)	1.41 (1.00,2.00)	2.179	0.029
ICV (ml; *M*, *IQR*)	21.00 (6.60,41.00)	9.10 (1.78,24.85)	27.60 (17.70,49.30)	−5.565	<0.001
Ischemic volume (ml; *M*, *IQR*)	173.40 (105.40,238.20)	150.10 (83.53,230.58)	185.50 (144.60,244.90)	−2.382	0.017
Mismatch (*M*, *IQR*)	148.4 (83.65,214.05)	130.40 (74.80,215.50)	158.70 (107.40,197.60)	−1.241	0.214
ICGR (ml/h; *M*, *IQR*)	3.50 (1.18,10.84)	1.44 (0.39,5.67)	5.39 (2.70,15.27)	−5.334	<0.001
Occlusion site				3.816	0.165
ICA	23 (15.0)	11 (15.7)	12 (14.5)		
MCA	105 (68.6)	52 (74.3)	53 (63.9)		
ICA + MCA	25 (16.3)	7 (10.0)	18 (21.7)		
Interval time (h; *M*, *IQR*)					
Onset-to-door	3.97 (1.76,7.47)	3.98 (1.93,7.51)	3.60 (1.50,7.43)	0.185	0.853
Onset-to-imaging	5.12 (2.66,8.33)	5.24 (2.91,8.25)	4.98 (2.00,8.65)	0.809	0.418
Onset-to-recanalization	8.30 (5.63,12.22)	8.33 (6.06,12.36)	8.23 (5.50,11.90)	0.401	0.688
IVT [*n* (%)]	51 (33.3)	24 (34.3)	27 (32.5)	0.053	0.818
Tandem [*n* (%)]	22 (14.4)	9 (12.9)	13 (15.7)	0.243	0.622
Stroke etiology [*n* (%)]				—	0.140^b^
LAA	88 (57.5)	47 (67.1)	41 (49.4)		
CE	46 (30.1)	16 (22.9)	30 (36.1)		
SOE	4 (2.6)	2 (2.9)	2 (2.4)		
SUE	15 (9.8)	5 (7.1)	10 (12.0)		

^a^1 mmHg = 0.133 kPa; ^b^Fisher’s exact test; *IQR*, Interquartile Range; *M*, Median; SBP, systolic blood pressure; DBP, diastolic blood pressure; NIHSS, National Institutes of Health Stroke Scale; ASPECTS, Alberta Stroke Program Early CT Score; ASITN/SIR, American Society of Interventional and Therapeutic Neuroradiology/Society of Interventional Radiology; ICV, Infarct core volume; Mismatch, (Ischemic volume—ICV); ICGR, Infarct core growth rate; ICA, Internal carotid artery; MCA, Middle cerebral artery; IVT, Intravenous thrombolysis; LAA, Large-artery atherosclerosis; CE, Cardiogenic embolism; SOE, Stroke of other determined etiology; SUE, Stroke of undetermined etiology.

### Multivariate logistic regression

**Figure 3:**
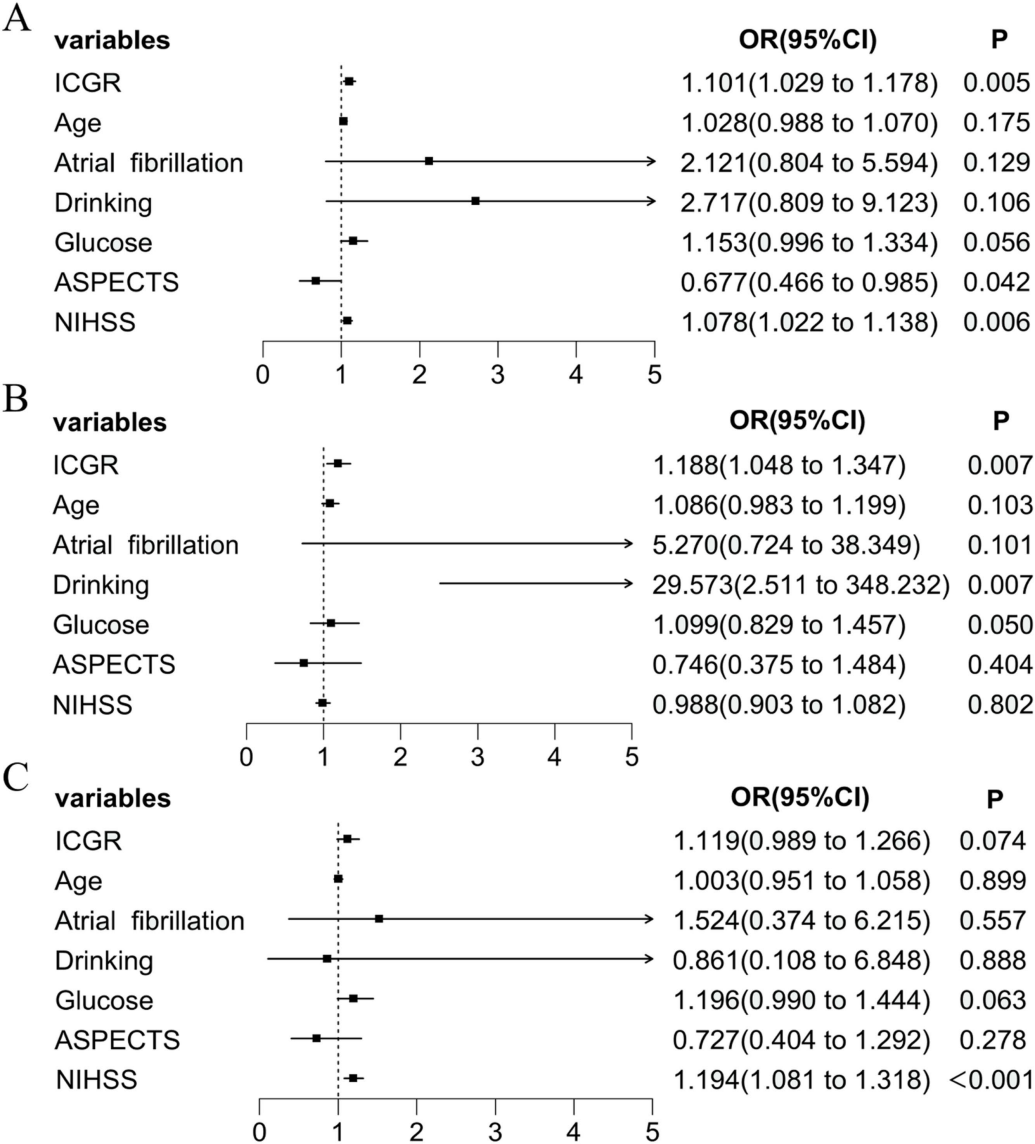
Forest plot of multivariate logistic regression analysis for poor prognosis. **(A)** Patients within 24 h of onset. **(B)** Patients within 8 h of ORT. **(C)** Patients beyond 8 h of ORT. ICGR, Infarct core growth rate; ASPECTS, Alberta Stroke Program Early CT Score; NIHSS, National Institutes of Health Stroke Scale; ORT, Onset-to-recanalization time.

The variables selected by Lasso regression were incorporated into the multivariate logistic regression analysis. The results showed that ICGR (OR 1.101, 95% CI 1.029–1.178; *p* = 0.005), ASPECTS score (OR 0.042, 95% CI 0.466–0.985; *p* = 0.039), and preoperative NIHSS score (OR 1.078, 95% CI 1.022–1.138; *p* = 0.006) were independent risk factors for poor outcomes in patients with anterior circulation stroke (Figure 3A).

### Subgroup analysis with ORT

We divided the patients into two groups based on the median time from onset-to-recanalization time (ORT) of 8 h. We again used a multivariable logistic regression analysis, and found that the subpopulation with a time from ORT < 8 h had an independent effect on the 90d prognosis of AIS-ACLVO patients (OR 1.188, 95% CI 1.048–1.347; *p* = 0.007), as shown in Figure 3B. However, another group with ORT > 8 h, the ICGR no longer showed statistical significance (Figure 3C).

**Figure 4:**
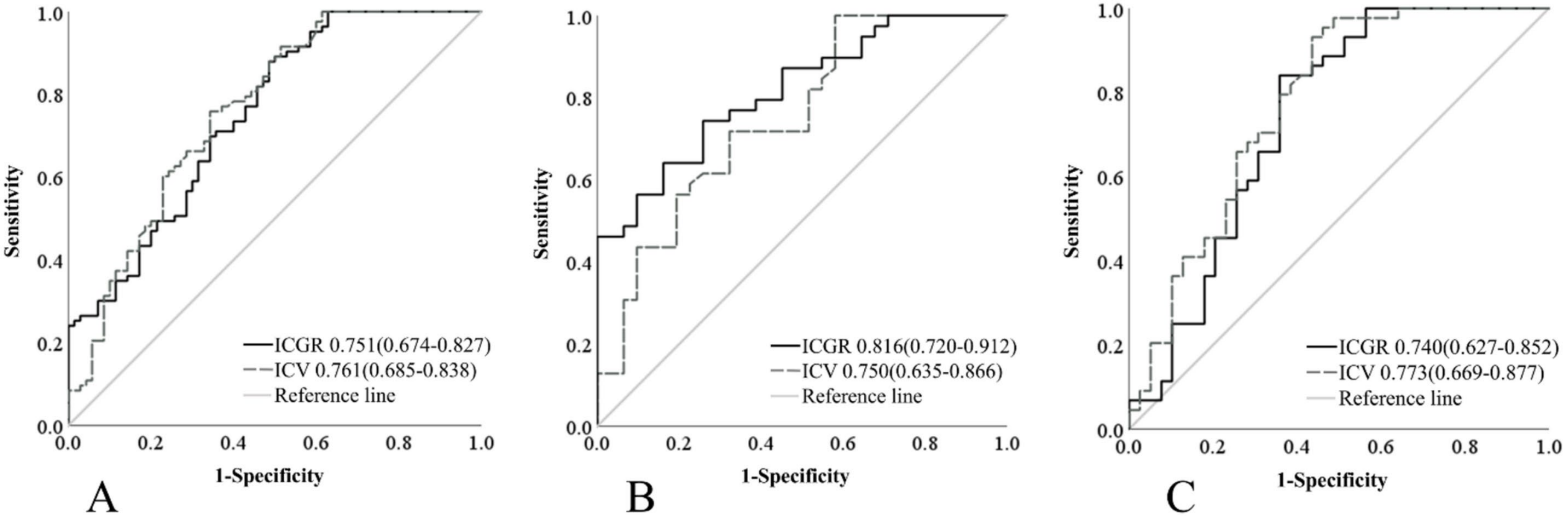
Comparison of ROC curves for ICGR and ICV among different groups. **(A)** Patients within 24 h of onset. **(B)** Patients within 8 h of ORT. **(C)** Patients beyond 8 h of ORT. ROC, Receiver operating characteristic; ICGR, Infarct core growth rate; ICV, Infarct core volume; ORT, Onset-to-recanalization time.

A single-factor logistic regression analysis was performed on the ICGR and ICV for different groups, and ROC curves were drawn and the AUC results were compared to show that in the subgroup with a time from ORT of 8 h or less, the AUROC curve for ICGR was higher than that for ICV (0.816 vs. 0.750), with a sensitivity of 74.4% and a specificity of 74.2%. Following the application of the Delong test for analytical purposes, a statistically significant disparity was observed between the two cohorts (*p* = 0.024), suggesting that the ICGR is a superior predictor of the 90d prognosis in AIS-ACLVO patients, within the 8-h window from ORT, in comparison with the ICV. No significant discrepancies were noted in the remaining groups (Table 2; Figure 4).

**Table 2:** Assess the predictive power of ICGR and ICV in various infarction subgroups.

Variable	All		≤8 h		>8 h	
	AUC	Delong	AUC	Delong	AUC	Delong
ICGR	0.751	*p* = 0.686	0.816	P = 0.024	0.740	*p* = 0.193
ICV	0.761		0.750		0.773	

### Comparison of ICGR and ICV

**Figure 5:**
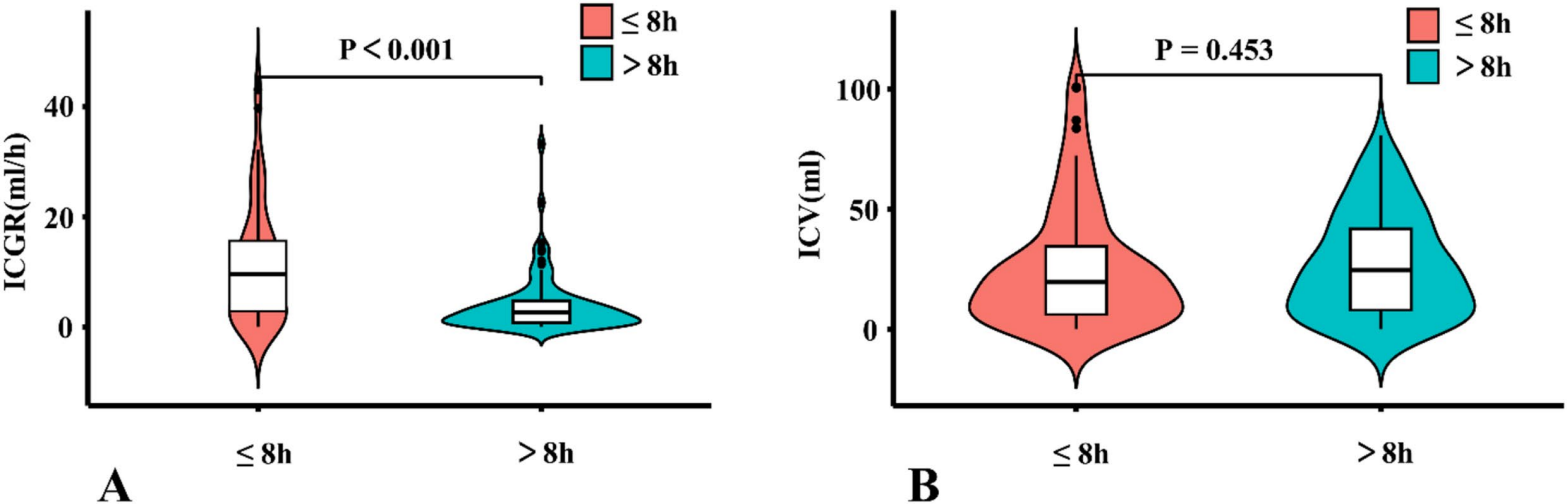
A violin plots comparing ICGR and ICV. **(A)** Comparison of the distribution and quartiles of ICV in patients. **(B)** Comparison of the distribution and quartiles of ICV in patients. ICGR, Infarct core growth rate; ICV, Infarct core volume.

There were a total of 70 (45.8%) patients with AIS-ACLVO who had a reperfusion time of 8 h or less, with the ICGR group having a median of 9.56 (IQR 2.67, 16.08) ml/h. Another group for patients with a reperfusion time of more than 8 h had a median of 2.64 (IQR 0.75, 4.77) ml/h, (*Z* = 4.601, *p* < 0.001), as shown in Figure 5A. The median ICV for patients with a reperfusion time of 8 h or less was 19.60 (IQR 6.1, 35.70) ml, while another group was 24.60 (IQR 7.70, 42.50) ml, (*Z* = −0.751, *p* = 0.453), as shown in Figure 5B.

## Discussion

Our study reports four key findings in patients eligible for thrombectomy: (1) Within 24 h of symptom onset, ICGR correlated significantly with 90-day clinical outcomes, with slower ICGR strongly associated with favorable prognoses. (2) ICGR independently predicted patient outcomes, demonstrating significant prognostic value within the first 8 h but no statistical significance beyond this time window. (3) Within the 8-h timeframe, ICGR exhibited stronger predictive efficacy for clinical outcomes compared to ICV. (4) ICGR was significantly higher within the first 8 h post-onset than after 8 h, whereas ICV did not differ significantly between these intervals.

The site of a cerebral infarction is commonly delineated into the infarct core and the ischemic penumbra (salvageable tissue). The magnitude of the infarct core and the ischemic penumbra tends to fluctuate in correlation with the progression of the infarction over time. The prolonged duration of infarction progression is positively correlated with an increase in the volume of the infarct core and a concomitant decrease in the extent of salvageable tissue. DEFUSE 3 trial found that EVT in patients with AIS-ACLVO and ICV measuring 70 mL or less within the first 16 h from the onset of symptoms often resulted in superior efficacy in terms of functional outcome endpoints (90d mRS score of 0–2) (3). Recent randomized controlled trials have demonstrated that EVT maintains clinical efficacy in patients with moderate-to-large ICV (70–100 mL) defined by NCCT and CTP (20–22). Notably, a study published last year in Lancet Neurology demonstrated that EVT in patients with AIS-ACLVO and large infarct core (ASPECTS score 3–5, corresponding to ICV < 100 mL) (23) within 12 h of symptom onset resulted in superior functional outcomes, survival rates, and quality of life compared to medical management alone at 12-month follow-up. These findings highlight the short-term and durable benefits of EVT in this population (24). Nonetheless, there is a paucity of reported research on the efficacy of thrombectomy for core infarctions exceeding 100 mL. In this study, there were 144 patients with ICV of 0–70 mL, accounting for approximately 94.12%, while others of 70–100 mL, accounting for 5.88%. The size of the ICV is related to the clinical prognosis of the patient. The larger the infarction core volume, the worse the prognosis of the patient (25–27). Our research further supports this view. However, in clinical practice, we have found that for patients with identical infarction volumes, yet shorter onset intervals, may have poor 90d outcomes (mRS scores of 3–6) after EVT, so we introduced the concept of ICGR. Prior research has demonstrated that the median growth rate of the infarcted core was in the range of 3–7 mL/h, with rapid progression of the infarcted core being delineated as an expansion exceeding 5 mL/h (13). Our dataset revealed a median growth rate of the infarcted core at 3.50 mL/h, and the majority of patients exhibiting unfavorable prognoses demonstrated rapid progression of the infarcted core, with a median value of 5.39 mL/h. Furthermore, our analysis indicated that patients with a more rapid progression of the infarcted core were of advanced age, presented with higher NIHSS score, and exhibited reduced ASPECTS score, aligning with the findings of prior research studies (14, 28).

Within a 24-h window following symptom onset, the ICGR not only significantly influenced the clinical outcomes of patients with AIS-ACLVO, but it also correlated with the patients’ functional recovery at the 90d outcome, even after adjusting for conventional prognostic variables such as age, baseline glucose levels, history of drinking, history of atrial fibrillation, ASPECTS score, and NIHSS score. An increase of 1 mL/h in the rate of infarct core expansion is associated with a 10.2% rise in the likelihood of an unfavorable outcome. Nonetheless, in univariate analysis, there is no statistically significant discrepancy in its prognostic impact when compared to the ICV.

We categorized the data based on the median ORT of 8 h. The findings suggested that, in patients with an onset-to-reopening time of 8 h or less, the ICGR served as a more reliable predictor of clinical outcomes compared to ICV. In the initial stages of a cerebrovascular accident, the brain parenchyma demonstrates a degree of tolerance to ischemic and hypoxic conditions. It is observed that a higher infarction ratio correlates with a diminished capacity for collateral circulation compensation, thereby implying that the penumbra—an area of potentially salvageable brain tissue—is at a greater risk of progressing to irreversible ischemic damage, even with timely interventions aimed at restoring cerebral blood flow (14, 15). Although the ischemic core region will continue to grow for at least 72 h after stroke onset (24), ICGR no longer independently predicts patient outcomes when the ORT exceeds 8 h. In this scenario, ICGR demonstrates no significant predictive superiority over ICV for clinical outcomes. Moreover, although our data showed no significant differences, existing literature suggests that ICGR may be related to the location of vascular occlusion, and occlusion of the internal carotid artery can predict ICGR (29). Proximity of the affected vessel to the distal extremity correlates inversely with the perfusion area’s size; hence, the penumbral tissue is more rapidly perfused by collateral circulation from the pial surface, which in turn retards the growth of the infarct core. Consequently, patients are more likely to exhibit enhanced functional independence 90 days subsequent to the incident.

When the duration of ORT exceeds 8 h, the ICGR no longer independently affects the prognosis of patients. At this point, the ICGR is no more advantageous than the ICV in predicting the clinical outcome of patients. Interestingly, we observed that the median ICGR for patients treated within 8 h of ORT was 9.56 mL/h, while for another subgroup only 2.64 mL/h, indicating a significant difference. However, no notable difference in ICV was observed between the two groups. This phenomenon may be explained by the fact that the maximum allowable ICV, as stated in the thrombectomy eligibility criteria, is 100 mL. In instances where the ORT is protracted and the rate of infarction is expedited, the infarcted volume in these patients significantly surpasses the 100 mL threshold, precluding them from thrombectomy within the framework of the EVT. By this time, the majority of brain tissue has suffered from extended ischemia, and the cumulative impact of the ICV becomes more pronounced. Consequently, for patients with AIS-ACLVO, the volume of infarction imposes constraints on the investigation of rapid ICGR.

The limitations of this study include the following aspects: (1) The sample size for this study is relatively limited, and there is a need to increase the sample size for more comprehensive analysis; (2) Our investigation constitutes a single-center study, with the potential for subsequent external validation to corroborate the findings; (3) This investigation omitted individuals exhibiting ICV exceeding 100 mL, particularly those with an expedited expansion of the infarcted core. Subsequent prospective clinical trials are imperative to address the management of these patients in the future.

## Conclusion

Our study findings suggest that the ICGR and the ICV measured within 24 h of onset in AIS-ACLVO patients are closely related to the 3-month outcomes. Within 8 h of ORT, we can predict the prognosis of patients by measuring the ICGR. This information can assist doctors in implementing more effective and rational treatment measures, ultimately helping patients achieve better clinical outcomes.

## Data Availability

The raw data supporting the conclusions of this article will be made available by the authors, without undue reservation.
